# Career perspectives of senior dental students from different backgrounds at a single Middle Eastern institution

**DOI:** 10.1186/s12909-018-1386-9

**Published:** 2018-11-26

**Authors:** Mohammad S. Alrashdan, Melanie Alazzam, Mustafa Alkhader, Ceib Phillips

**Affiliations:** 1 Department of Oral Medicine and Oral Surgery, Faculty of Dentistry, Jordan University of Science and Technology, P.O.Box 3030, Irbid, 22110 Jordan; 2 Department of Orthodontics, School of Dentistry, University of North Carolina, Chapel Hill, NC USA

**Keywords:** Career plans, Dental students, Socioeconomic, Middle East

## Abstract

**Background:**

Differences between dental students in terms of social, economic and cultural backgrounds are likely to impact their professional career plans. The aim of this study was to explore the professional career plans among final year dental students from different backgrounds at a single Middle Eastern institution (Jordan university of Science and Technology-JUST).

**Methods:**

Fifth year dental students at JUST were invited to fill out a paper based self-administered questionnaire. Data was collected on students` demographics, their future career plans and the impact of social and economic changes on such plans, their interest in postgraduate studies and the specialty of choice in addition to the influence of a group of factors on that choice. Data was also collected on the value of non-academic workshops, guidance regarding career plans, participants` preferred pattern of work (full-time versus part-time) and retirement plans. Students were categorized according to their nationalities. Pearson’s chi squared test, one way ANOVA and post hoc tests were used to measure statistical significance between measured variables and backgrounds of participants. The level of significance was set at *P* ≤ 0.05.

**Results:**

A total of 227 students completed the survey (response rate = 84%). 47% of the participants were Jordanians, 27% were Malaysians, 11% were from Gulf States (Saudi Arabia, Bahrain, Kuwait and Qatar), 10% were from conflict zones in the Middle East (Syria, Iraq, Palestine and Yemen) and 5% comprised students from other nationalities. Significant differences were found between students from different backgrounds in their funding sources (Chi square = 132, *P* < 0.01), practice plans (Chi square = 43, P < 0.01), the impact of social and economic changes on their career choices (Chi square = 34, *P* < 0.01), planned work pattern within the first 10 years post-graduation (chi square = 18, *P* < 0.05) and 10–20 years after graduation (69%, Chi square = 22, P < 0.01) and retirement plans (Chi square = 25, *P* < 0.05). Students from different nationalities agreed on most factors affecting their choice of a specialty, except for the reputation of the specialty (P < 0.05).

**Conclusion:**

Several differences in career plans were found between dental students from variable backgrounds studying a single institution. Many of these disparities could reflect variations in socioeconomic backgrounds.

**Electronic supplementary material:**

The online version of this article (10.1186/s12909-018-1386-9) contains supplementary material, which is available to authorized users.

## Background

Oral healthcare providers, primarily dentists, represent an integral part of the healthcare workforce [1, 2]. As political and socioeconomic factors are major determinants of healthcare policies and plans, these factors are inherently pivotal in shaping the future of professions and career perspectives for students and trainees in the healthcare domain [3, 4]. A position paper published in 2012 has outlined three possible scenarios for the future of dental practice and education and linked each to specific socioeconomic predictions [5]. The scenarios were simply referred to as optimistic, moderate and pessimistic. Although these anticipated scenarios were relating to dentistry within the USA, they could be applied to dental practice in all nations, particularly those with unstable conditions. In a region like the Middle East, where major geopolitical instability and dramatic socioeconomic changes have been taking place in many countries, the pessimistic scenario would be highly predicted. The key aspects of this scenario include a subtle increase in dental expenditure and fewer resources to be directed to dentistry and oral healthcare [5], which can be directly attributed to the economic recession that usually accompanies political and military conflicts .

Despite being in the middle of a volatile region, Jordan has managed to maintain a relatively stable political and social status, and provided a reputable yet affordable alternative to students from unstable neighbouring countries (particularly Syria, Iraq, Yemen and Palestine) seeking to major in dentistry but cannot do so in their homelands. Apart from Middle Eastern unstable countries, Jordan has also provided a sound choice for students from the wealthier, more stable Gulf countries (Saudi Arabia, Bahrain, Kuwait and Qatar) who wish to study dentistry in a nearby country that can provide accredited dental programs within a cultural environment similar to their own countries. The faculty of dentistry at Jordan University of Science and Technology (JUST) is one of the two in the country with 1678 undergraduate and 76 graduate students from different Middle Eastern and Asian (mainly Malaysian) and other nationalities ([http://www.just.edu.jo/FacultiesandDepartments/FacultyofDentistry/Pages/Highlights.aspx]).

Previous reports on career plans for dental students in the Middle East have shown that private practice was a preferred choice in some countries (e.g. Iran and Turkey) [6, 7] but not the others (Saudi Arabia) [8],while a majority of students from several countries have shown an interest in postgraduate studies with some variations in most desired specialties [9–11]. However, none of these studies have compared career perspectives between students from different backgrounds or addressed the future career intentions in terms of work pattern or retirement plans among participants. Therefore, the current study was designed to describe and compare the professional career plans among final year dental students from different backgrounds at a single Middle Eastern institution (JUST) and to highlight factors that affect such professional plans.

## Methods

This study was approved by JUST Institutional Review Board (IRB), approval reference number 390/2017. Fifth year dental students at JUST were invited to fill out a paper-based questionnaire (Additional file 1- survey). The questionnaire was pretested for clarity of questions and adequacy of response options on a cohort of 25 students.

The questionnaire items were explained to students, stating clearly that participation was completely voluntary, anonymous and would not affect the students` academic grades in any manner.

The structured questionnaire was composed of two major sections. The first section was designed to collect the participant’s demographic details (age, gender, marital status, nationality, tuition fee source and parental educational level). The second section included a set of 11 questions (ten multiple choice items and one likert scale item) that explored students` career intentions (supplemental material.

Students were first asked about their future career plan in general (i.e. private, public, academic, research and others). Students were then questioned, using a Likert scale, about their perspectives on the impact of social and economic changes within their respective communities on their career choices. Next, students were asked about their interest in postgraduate studies and the program structure they prefer, in addition to their specific dental specialty of choice. Thereafter, students were asked to select a degree of influence (from 1 to 5) for several factors on their specialty choice. The factors were; personal desire, financial motives, reputation of specialty, length of training, affordability of tuition fees, future flexibility in work, low stress and others` influence. Data was also collected on the value of non-academic workshops and courses and whether students think these could be an alternative to classic academic programs or not. Students were then asked if they have received guidance with regards to their career plan and the source of the guidance if present. Finally, participants were asked about the pattern of work (full-time versus part-time) they prefer within the first ten years, ten to twenty years after graduation and thereafter, as well as their planned age of retirement.

For data analysis purposes, students were grouped, according to their nationalities, into one of five groups; Jordanians, Malaysians, Gulf States (Saudi Arabia, Bahrain, Kuwait and Qatar), active conflict zones (Syria, Iraq, Palestine and Yemen) and others.

SPSS software package Version 20.0 (SPSS Inc., Chicago, IL, USA) was used for statistical analysis (frequency distribution and inferential analysis). Pearson’s chi squared test was used to determine the significance of relationship between collected data and the nationalities of participants. One way ANOVA and post hoc tests was used to compare the factors influencing the specialty choice between nationalities (section two, question six). The level of significance was set at *P* ≤ 0.05.

## Results

Out of 270 students, a total of 227 students completed the survey (response rate = 84%). Their demographical details are shown in Table 1. About half of the participants were Jordanians (47%), while Malaysians, Gulf States and areas of conflict students represented 27, 11 and 10%, respectively. The remainder 5% comprised students from other nationalities including other Asian and African backgrounds. Females represented 73% of the study population and because the dental program at JUST is a bachelor’s (BDS) rather than a doctorate degree (DDS), the vast majority of students were younger than 25 years (98%) and single (92%).

**Table 1:** Demographic details of a group of senior dental students at Jordan University of Science and Technology (*n* = 227)

*Characteristic*	*Nationality*					*Total* n(%)
	Jordanians	Malaysians	Gulf States	Syrians, Iraqi and Palestinians	Others	
Gender						
Female	81 (76%)	48 (77%)	15 (60%)	15 (68%)	7 (64%)	166 (73%)
Male	26 (24%)	14 (23%)	10 (40%)	7 (32%)	4 (36%)	61 (27%)
Age						
≤ 25	106 (99%)	62 (100%)	22 (88%)	21 (96%)	11 (100%)	222 (98%)
> 25	1 (1%)	0	3 (12%)	1 (4%)	0	5 (2%)
Marital status						
Single	101 (94%)	60 (97%)	20 (80%)	18 (82%)	11 (100%)	210 (92%)
Married	6 (6%)	2 (3%)	5 (20%)	4 (18%)	0	17 (8%)
Tuition fee source*						
Own or parental	87 (81%)	1 (2%)	10 (40%)	21 (96%)	5 (46%)	124 (55%)
Scholarship	19 (18%)	54 (87%)	9 (36%)	1 (4%)	6 (54%)	89 (39%)
Loan or other	1 (1%)	7 (11%)	6 (24%)	0	0	14 (6%)
Father’s educational level						
< high school	4 (4%)	0	0	0	2 (18%)	6 (3%)
High school	8 (8%)	14 (23%)	8 (32%)	4 (18%)	1 (9%)	35 (15%)
Higher education (undergraduate or postgraduate)	95 (88%)	47 (77%)	27 (68%)	18 (82%)	8 (73%)	186 (82%)
Mother’s educational level						
< high school	3 (3%)	0	5 (20%)	0	1 (9%)	9 (4%)
High school	13 (12%)	16 (26%)	5 (20%)	6 (27%)	4 (36%)	44 (19%)
Higher education (undergraduate or postgraduate)	91 (85%)	46 (74%)	15 (60%)	16 (73%)	6 (55%)	174 (77%)
Subtotal	107 (47%)	62 (27%)	25 (11%)	22 (10%)	11 (5%)	227

*Significant differences between nationalities, Chi square test, *P* < 0.05

Students from different nationalities were found to have a statistically significant difference in funding sources (Chi square = 132, *P* < 0.01). While a majority of Jordanian and conflict zones students (81 and 96%, respectively) were self or parental supported, most Malaysians were on a scholarship (87%). Funding for Gulf States` students was distributed between self (40%), scholarships (36%) and loans (24%). The parents` educational level for all nationalities indicated that most students came from highly educated families where both parents hold either an undergraduate or a postgraduate degree without significant differences between nationalities (*P* > 0.05).

In terms of practice plan, more Jordanian and Syrian, Iraqi and Palestinian students preferred to have a career in the private sector (46 and 77% respectively) as shown in Table 2, while the public domain was a more attractive choice for Malaysians and Gulf States students (Chi square = 43,*P* < 0.01). A common finding among all nationalities was that a career in research was not attractive and was considered by only four Malaysian students and a single Jordanian student.

**Table 2:** Practice plans and related factors, interest in postgraduate studies, preferred program structure and specialty of interest, views on workshops and guidance regarding career plans among a group of senior dental students at Jordan University of Science and Technology (n = 227)

*Characteristic*	*Nationality*					*Total* n(%)
	Jordanians	Malaysians	Gulf States	Syrians, Iraqi and Palestinians	Others	
Practice plan*						
Private	49 (46%)	15 (24%)	4 (16%)	17 (77%)	4 (36%)	89 (39%)
Public	30 (28%)	33 (52%)	16 (64%)	3 (13%)	6 (55%)	88 (38%)
Academic	23 (21%)	9 (14%)	5 (20%)	1 (5%)	1 (9%)	39 (17%)
Research	1 (1%)	4 (7%)	0	0	0	5 (2%)
Others	4 (4%)	1 (3%)	0	1 (5%)	0	6 (3%)
Impact of social and economic changes on practice plan*						
Strong	49 (46%)	22 (36%)	5 (20%)	11 (50%)	2 (18%)	89 (39%)
Moderate	44 (41%)	34 (55%)	1 (4%)	7 (31%)	7 (64%)	93 (41%)
Weak	5 (5%)	6 (9%)	13 (52%)	3 (14%)	2 (18%)	29 (13%)
None	9 (8%)	0	6 (24%)	1 (5%)	0	16 (7%)
Interest in PG studies						
Yes	91 (86%)	52 (84%)	21 (84%)	19 (86%)	8 (73%)	191 (84%)
No	6 (5%)	2 (3%)	0	2 (9%)	0	10 (4%)
Undecided	10 (9%)	8 (13%)	4 (16%)	1 (5%)	3 (27%)	26 (12%)
Preferred PG structure						
Clinical	53 (58%)	36 (69%)	16 (76%)	14 (73%)	6 (75%)	125 (66%)
Research	3 (3%)	2 (4%)	1 (5%)	0	0	6 (3%)
Combined	31 (34%)	10 (19%)	3 (14%)	3 (16%)	1 (12%)	48 (25%)
Undecided	4 (4%)	4 (8%)	1 (5%)	2 (11%)	1 (12%)	12 (6%)
Specialty of interest						
Restorative dentistry	23 (25%)	11 (21%)	10 (47%)	5 (26%)	4 (50%)	53 (28%)
Prosthodontics	20 (22%)	4 (8%)	1 (5%)	1 (5%)	0	26 (13%)
Orthodontics	16 (18%)	2 (4%)	1 (5%)	4 (21%)	1 (12%)	24 (12%)
Periodontics	6 (7%)	9 (17%)	3 (14%)	2 (11%)	2 (25%)	22 (12%)
Oral surgery	13 (14%)	8 (15%)	1 (5%)	0	0	22 (12%)
Paedodontics	2 (2%)	9 (17%)	1 (5%)	3 (16%)	0	15 (8%)
Others^a^	5 (5%)	8 (15%)	3 (14%)	1 (5%)	0	17 (9%)
Undecided	6 (7%)	1 (2%)	1 (5%)	3 (16%)	1 (12%)	12 (6%)
Non academic courses/workshops as alternatives to PG programs						
Yes	27 (25%)	23 (37%)	5 (20%)	10 (45%)	3 (27%)	68 (30%)
No	67 (62%)	26 (42%)	17 (68%)	8 (36%)	4 (36%)	122 (54%)
Undecided	13 (13%)	13 (21%)	3 (12%)	4 (19%)	4 (36%)	37 (16%)
Guidance regarding career plan provided						
Yes	29 (27%)	8 (13%)	9 (36%)	7 (32%)	4 (36%)	57 (25%)
No	78 (73%)	54 (87%)	16 (64%)	15 (68%)	7 (74%)	170 (75%)
Guidance source						
Dental faculty	12 (41%)	3 (38%)	2 (22%)	1 (14%)	1 (25%)	19 (34%)
National Dental association	6 (21%)	1 (12%)	0	3 (44%)	1 (25%)	11 (19%)
National Student association	6 (21%)	2 (25%)	7 (78%)	1 (14%)	0	16 (28%)
Others	5 (17%)	2 (25%)	0	2 (28%)	2 (50%)	11 (19%)

Chi square test,*P < 0.05

^a^Other specialties include: oral medicine, oral radiology, oral pathology, dental basic sciences

When students were asked about the impact of social and economic changes within their communities on their career choices, statistically significant differences were found (Table 2) (Chi square = 34, *P* < 0.01). Almost half of students from communities facing major challenges (Jordanians in one group, Syrians Iraqi and Palestinians in the other) acknowledged that the impact was strong, whereas about half of students from Malaysia thought that these changes were only moderate in importance. Interestingly, three quarters of Gulf States` students believed that such changes are too subtle to influence their professional plans.

Interest in postgraduate studies in dentistry and a preference of a clinical program were not statistically different between nationalities (*P* > 0.05). Moreover, attractive specialties to students were similar across nationalities as shown in Table 2. Students from different nationalities agreed on most factors affecting their choice of a specialty, except for the reputation of the specialty as shown in Table 3, where students from conflict zones considered reputation as a strong influential factor affecting their specialty choice (*P* < 0.05).

**Table 3:** Factors influencing the specialty of choice, expressed as the median for each nationality, among a group of senior dental students at Jordan University of Science and Technology (n = 227)

*Factor*	*Nationality*				
	*Jordanians*	*Malaysians*	*Gulf states*	*Syrians, Iraqi and Palestinians*	*Others*
Personal desire	4(strong)	4(strong)	4(strong)	5(strong)	4(strong)
Financial motives (anticipated income)	3(moderate)	3(moderate)	4(strong)	4(strong)	3(moderate)
Reputation of specialty*	3(moderate)	3(moderate)	3(moderate)	4(strong)	3(moderate)
Length of training	3(moderate)	3(moderate)	4(strong)	4(strong)	3(moderate)
Affordability (tuition/training fee)	4(strong)	3(moderate)	4(strong)	3(moderate)	4(strong)
Flexible working time	3(moderate)	4(strong)	3(moderate)	3(moderate)	3(moderate)
Low stress level	3(moderate)	4(strong)	3(moderate)	3(moderate)	3(moderate)
Other’s influence	2(weak)	3(moderate)	2(weak)	2(weak)	2(weak)

One way ANOVA and post hoc tests,*P < 0.05

Non-academic courses and workshops were not considered as satisfactory alternatives to academic postgraduate courses by most students. Results also showed that only 25% of students had received some sort of guidance regarding their career plans at this stage.

With regards to planned work pattern, statistically significant differences among nationalities (Table 4) were found. A vast majority of Malaysian students (87%) planned for a full time career in their first 10 years post-graduation, compared to 62%,72 and 68% of Jordanian, Gulf States and conflict zones` countries, respectively (chi square = 18, *P* < 0.05). The proportion of students planning for a full-time job 10–20 years post graduation, declined for all nationalities compared to the first 10 years, while Malaysians maintained the highest proportion in this respect compared to others (69%, Chi square = 22, *P* < 0.01). Thereafter, students showed no differences in their planned work pattern following 20 years after graduation, where most of them opted for a part-time career or were indecisive about this stage.

**Table 4:** Planned employment pattern and retirement age among a group of senior dental students at Jordan University of Science and Technology (n = 227)

*Work pattern*	*Nationality*					*Total n(%)*
	*Jordanians*	*Malaysians*	*Gulf states*	*Syrians, Iraqi and Palestinians*	*Others*	
First 10 years*						
Full time	66 (62%)	54 (87%)	18 (72%)	15 (68%)	10 (90%)	163 (72%)
Part time	31 (29%)	5 (8%)	4 (16%)	6 (27%)	0	46 (20%)
Undecided	10 (9%)	3 (5%)	3 (12%)	1 (5%)	1 (10%)	18 (8%)
10–20 years**						
Full time	38 (35%)	43 (69%)	6 (24%)	9 (41%)	5 (45%)	101 (44%)
Part time	48 (45%)	9 (15%)	8 (32%)	9 (41%)	5 (45%)	79 (35%)
Undecided	21 (20%)	10 (16%)	11 (44%)	4 (18%)	1 (10%)	47 (21%)
After 20 years						
Full time	11 (10%)	19 (30%)	2 (8%)	5 (23%)	4 (36%)	41 (18%)
Part time	47 (44%)	27 (44%)	10 (40%)	10 (45%)	5 (45%)	99 (44%)
Undecided	49 (46%)	16 (26%)	13 (52%)	7 (32%)	2 (19%)	87 (38%)
Planned age of retirement*						
< 50 years	18 (17%)	5 (8%)	7 (28%)	2 (9%)	1 (10%)	33 (15%)
50–60 years	30 (28%)	36 (58%)	5 (20%)	5 (23%)	4 (36%)	80 (35%)
> 60 years	33 (31%)	13 (21%)	8 (32%)	10 (45%)	3 (27%)	67 (30%)
Undecided	26 (24%)	8 (13%)	5 (20%)	5 (23%)	3 (27%)	47 (21%)

Chi square test, *P < 0.05, **P < 0.01

Finally, differences were noticed between students in terms of their respective retirement plans (Chi square = 25, *P* < 0.05), Table 4. Collectively, two thirds of students planned for retirement after the age of 50.

## Discussion

Regular monitoring of career intentions for students in the healthcare domain is of paramount importance in the planning process for healthcare systems worldwide. Literature search has shown a paucity of such reports in relation to dental students within the Middle East (with the exception of Turkey). Therefore, the value of the current report is on one hand being an addition to the very few reports from the Middle Eastern countries, and on the other hand the fact that it involves students from different backgrounds, with widely variable socioeconomic conditions that may affect their career perspectives.

Funding for Jordanian students at JUST relies mainly on parental financial support, a finding that is supported by a previous report at the University of Jordan [12]. In Jordan, parents traditionally support their children’s primary and secondary education and support post-secondary education as well. Non-Jordanian students, mainly those from Malaysia and Gulf states, are admitted at JUST through agreements signed between JUST and the cultural attaché of these countries. In such agreements, the cultural attaché of these countries cover the tuition fee and living expenses of their respective students (personal communication: JUST international students’ office). This explains the fact that greater proportion of Malaysian and Gulf state students depend on scholarships as a funding source at JUST.

The high level of parental educational background of our students` population is consistent with previous reports where family socioeconomic status had a major role in students’ educational attainment and access to higher education [11, 13–15]. This issue has gained attention where professional and government initiatives were recommended in order to attract applicants from a wider range of social and school backgrounds so that the access to higher education (dentistry in our case) is not limited to selected advantaged groups of the community [14].

The variations in practice plans between different nationalities could be at least partially attributed to different socioeconomic conditions at students` homelands. While Gulf States and Malaysia are endowed with relatively stable economic systems, these countries are more able to create and maintain jobs within the public sector, while providing financial stability for employees. This notion is supported by reports from the UAE and Saudi [8, 10]. As for Malaysia, although working in the private sector offers dentists greater financial rewards, it was reported that most dentists prefer to work in the public sector where they could access other benefits, such as scholarships for postgraduate training [16]. In line with these findings and at a wider scale, an improvement in oral health care provision, delivered through public services, is witnessed in countries exhibiting a relative political stability and programmed economic growth, such as India, Brazil, Russia and China [17]. Interestingly, a Brazilian study has shown that most students plan to become specialists and work in both the public and private sectors simultaneously [13]. Such finding was explained by the desire to achieve both financial stability and job security. On the other hand, private practice will be a more sound choice when social and economic instability prevail, such as in Iraq, Syria, Palestine and even Jordan. In addition to more financial satisfaction compared to public domain, private practice provides a more flexible career choice where a dentist could practice at different places within the same country or easily change practice location locally or abroad. A report from Iran reflects the same attitude where private practice is desired by most dental students [6].

Lack of interest in research jobs,as reported in the current study, appears to be common among dental students regardless of their nationalities [18]. This particular finding was further supported by the high interest in clinical postgraduate programs within our population, compared to combined or research-based programs.

This could be explained by several factors including the personal desire aspect where most dental students would like to practice clinical dentistry as opposed to a research career, as well as the financial motives. The urgent need of more focus on research training and scholarship to be introduced and reinforced within the environment and culture of dental schools and programs has been recently acknowledged as a part of a project aiming at advancing dental education in the twenty-first century and ensuring the future standing of the dental profession [19]. Therefore, reengineering of dental training programs to improve and maintain the research capacity of oral health researchers is deemed essential for the preparation of future graduates for team science, clinical trials, and translational research as well as other emerging opportunities [19, 20].

It is generally accepted that career choices are based on a combination of interests and aptitude of the individuals as well as familial and environmental influences. Several authors provided models to highlight the importance of socioeconomic factors in career planning [21],however, the impact of such variables on the occupational choices of individuals has not always been well addressed [22] .We have attempted to highlight this aspect in the current study and found significant differences between students from different backgrounds and socioeconomic conditions.

The marked interest in postgraduate studies in our population did not deviate from previous reports within the Middle East. For instance, Khami et al. reported a high rate (70%) of interest in postgraduate studies within Iranian senior dental students surveyed nationwide [23]. Moreover, Tanalap et al. found that 86% of undergraduate students enrolled in a Turkish dental school had an interest in pursuing a specialty degree in dentistry [11]. Within the Gulf States, Halawany et al. reported that 96% of students surveyed at several Saudi dental schools expressed interest in postgraduate studies [8]. Similarly, in a report from the UAE, 92% of participating students had a plan for a dental specialty after graduation from the dental school [10].

Furthermore, restorative dentistry took the lead as the specialty of choice among the others. Prosthodontics, periodontics, orthodontics and oral surgery ranked next at almost the same level. A review of literature in this aspect reflects a marked interest to specialise in orthodontics as the first choice for most students (excluding those who were undecided in this respect) surveyed in several reports from different regions, including Europe, North America, Asia and the Middle East [10, 11, 18, 24–27]. Our findings could indicate a change in such a classic trend, probably due to changes within the dental profession itself. However, further studies on a larger scale are required to confirm this notion.

Personal desire and affordability of specialty programs were the factors with the highest impact among our participants. Shin et al. reported that enjoyment of providing a given type of specialty service was the leading influential factor deciding the specialty choice for students at Harvard School of Dental Medicine surveyed at two independent occasions with a five year interval; 2008 and 2013 [26]. In the same study, the importance of the cost of the specialty program did not reach significance. Similarly, dental students surveyed in several Middle Eastern institutions based their specialty choice on a number of factors including personal characteristics and an interest in specific patient population [8, 23]. Therefore, it appears that personal views and perceptions are common factors in the process of deciding which specialty to choose among dental students. It is noteworthy that the reputation of the specialty was significantly different in terms of importance to students based on their nationalities. Students from conflict zones believed that this factor has a strong influence on their choices compared to other students who thought this is only moderate in importance.

Participants` views on non-academic courses and workshops showed that most of them considered these to be a supplement, rather than an alternative to classic academic courses. This is probably due to the fact that such courses are usually conducted in a short time frame and this will result in insufficient knowledge and practice compared to academic programs. In one report that questioned the effectiveness of short dental courses in improving the skills and knowledge of targeted dental personnel, such courses were found unsatisfactory and the prescribed standard was achieved by only 60% of participants [28]. Moreover, it has been documented that a majority of practicing dentists would prefer modular programs leading to a postgraduate qualification over short term courses [29] and that their continuing professional development (CPD) activities are influenced by the requirements of external regulatory bodies and not necessarily to improve their knowledge or practice [30].

Our results indicate that career advice to senior dental students is still unsatisfactory. Such guidance to dental students should be based on communities` needs and respective oral healthcare plans and this cannot be over emphasized and has been highlighted both regionally [8] and internationally [31]. More efforts should be made in this direction by respective national dental associations in collaboration with dental schools.

Finally, with regards to work pattern and retirement plans, our participants have shown some variations in their perspective. A majority of dental students surveyed in different reports have indicated an interest in a full time job immediately after graduation with an overall inclination towards a part time job after being in practice for a while (5–15 years, variable between studies) [10, 24, 27]. This plan was also noticed among our participants.

Previous reports have shown some variations in planned retirement age among dental students or dentists, sometimes even within the same nation. Stewart et al. reported that 59% of a cohort of UK dental students intended to retire before the age of 60 [32], while Puryer and Patel found a much less percentage of students (38%) with the same early retirement plan in another more recent study within the UK [24]. The latest authors proposed that this wide difference could be due to local factors relevant to pension regulations or an increased students` perception of debt. In one study from Lithuania, more than 65% of practicing dentists and dental specialists surveyed had plans to work after retirement age (i.e. after the age of 60). Socioeconomic circumstances may again help to explain the variations noticed within our population. For example, Gulf States` students scored the highest percentage in the category of those planning to retire before the age of 50, and this could be the result of an anticipation that financial security could be achieved at a younger age within prosperous economies, as opposed to students from conflict zones who scored the highest percentage in those wishing to retire after the age of 60. Collectively, about two thirds of students participating in this study have chosen a reasonable retirement age (above 50), which indicates an overall appreciation of the changing economic conditions across the globe where early retirement is not considered an affordable choice.

Shortcomings of the current study include the relatively small sample size, particularly from Gulf countries and areas of conflict within the Middle East, lack of participants from other universities and the fact that students from all backgrounds were residing in Jordan for most of the academic year over the past five years, so their perception of the most recent socioeconomic changes within their respective communities might be inadequate.

## Conclusions

The current study has highlighted several differences in career plans between dental students from variable backgrounds studying a single institution. Many of these disparities could reflect variations in socioeconomic conditions of the students. Stable and programmed economic growth makes public practice more appealing compared to unstable socioeconomic circumstances, where a career in private practice becomes a safer option. A marked interest in clinical dentistry and postgraduate studies is still very popular among students; however, there might be a change in terms of specialties in high demand. Personal desire and affordability are the key determinants of the specialty of choice. A full-time career is an acknowledged necessity by a growing number of students especially at the beginning of their careers and early retirement is not an option for most of them. Our findings should be confirmed by further focused research on a wider scale including students from different universities. The reported practice plans, working pattern and retirement plans, however, could serve as a provisional reference for healthcare policy makers in the participating students` nations.

## Additional file

Additional file 1: A survey of career perspectives of senior dental students at Jordan University of Science and Technology. This file represents the questionnaire used in the current study. (DOCX 41 kb)

## Abbreviations

IRB: institutional research board
JUST: Jordan University of Science and Technology
