# Supplementary material for: Career perspectives of senior dental students from different backgrounds at a single Middle Eastern institution
Source: BMC Med Educ. 2018 Nov 26;18:283. doi: 10.1186/s12909-018-1386-9 (PMC6257955; doi:10.1186/s12909-018-1386-9)
Supplement: Supplementary file 1 — A survey of career perspectives of senior dental students at Jordan University of Science and Technology. This file represents the questionnaire used in the current study. (DOCX 41 kb) [file 12909_2018_1386_MOESM1_ESM.docx]

**A survey of career perspectives of fifth year dental students at Jordan University of Science and Technology**

**Part One (General Information and Demographics)**

1. Age: ○ ≤25 ○>25

2. Gender: ○Male ○Female

3. Marital Status: ○Single ○Married

4. Nationality: ○Jordanian ○Malaysian ○Gulf States (Saudi, UAE, Kuwait, Bahrain Qatar, Oman) ○ Syrians, Iraqi, Palestinians ○Other: _________

6. Where does the **majority** of the money for your study come from (please select one only):

- Own or parental money
- Scholarship
- Loan or others

8. Father's educational level:

○Less than high school ○High school ○ Undergraduate level ○ Postgraduate level.

9. Mother's educational level:

○Less than high school ○High school ○Undergraduate level ○ Postgraduate level.

**Part Two (Career Intentions)**

1. What is your future practice plan?

- Private practice
- Public sector (governmental or armed forces)
- Academic institution
- Research facility
- Other (please specify)__________________________

1. What is the impact of recent social and economic changes within your country (if any) on your future career plan?

- Strong
- Moderate
- Weak
- None

1. Are you interested in pursuing studies after dental school (postgraduate studies)? If not or undecided, please skip to question 7.

- Yes
- No
- Undecided

1. What structure would you prefer for a postgraduate program?

- Clinical (i.e. specialty program)
- Research programs (e.g. PhD, MPhil, MSc)
- Combined programs (clinical + research)
- Undecided

1. What is your preferred specialty?

- Restorative dentistry
- Prosthodontics
- Orthodontics
- Periodontics
- Oral surgery
- Paedodontics
- Other, please specify_____________________________________
- Undecided

1. Among the following factors, please indicate the level of influence of each factor on your postgraduate specialty choice?

| Factor | Level of influence | | | | |
| --- | --- | --- | --- | --- | --- |
|  | weak | | moderate | strong | |
| Personal desire/ interest | 1 | 2 | 3 | 4 | 5 |
| Financial reasons/motives (future income) | 1 | 2 | 3 | 4 | 5 |
| Reputation of specialty | 1 | 2 | 3 | 4 | 5 |
| Length of training required | 1 | 2 | 3 | 4 | 5 |
| Affordability of tuition fees | 1 | 2 | 3 | 4 | 5 |
| Flexible working hours | 1 | 2 | 3 | 4 | 5 |
| Low stress level | 1 | 2 | 3 | 4 | 5 |
| Others` influence | 1 | 2 | 3 | 4 | 5 |

1. Do you think that non-academic training courses/workshops (including online courses) provide an adequate alternative to academic postgraduate programs? (non-academic courses/workshops are those courses ***not*** awarding an academic degree)

- Yes
- No
- Undecided

1. Have you been offered any help/guidance regarding you future career plans? (if your answer is (No) skip to question 10).

- Yes
- No

1. Who offered the guidance?

- Dental school Faculty
- National dental association
- Student association
- Other (please specify)

1. What pattern of work do you plan,

- Within the first 10 years after your final graduation from all training:
- Full time
- Part time
- Undecided
- 10-20 years of practice:
- Full time
- Part time
- Undecided
- After 20 years of practice:
- Full time
- Part time
- Undecided

1. At what age do you plan to retire?

- Below 50
- 50-60
- Above 60
- Undecided
